# Characteristics and severity of asthma in children with and without atopic conditions: a cross-sectional study

**DOI:** 10.1186/s12887-015-0481-x

**Published:** 2015-11-06

**Authors:** Ali Arabkhazaeli, Susanne J. H. Vijverberg, Francine C. van Erp, Jan A. M. Raaijmakers, Cornelis K. van der Ent, Anke H. Maitland van der Zee

**Affiliations:** Division of Pharmacoepidemiology & Clinical Pharmacology, Utrecht Institute for Pharmaceutical Sciences (UIPS), Faculty of Science, Utrecht University, P.O. Box 80082, David de Wied Building, Universiteitsweg 99, Utrecht, 3508 TB The Netherlands; Department of Pediatric Respiratory Medicine, Wilhelmina Children’s Hospital, University Medical Centre Utrecht, Lundlaan 6, Utrecht, 3584 EA The Netherlands

**Keywords:** Allergy, Asthma, Atopic condition, Eczema, Exacerbation, FeNO, Food allergy, Hay fever

## Abstract

**Background:**

Childhood allergic diseases have a major impact on a child’s quality of life, as well as that of their parents. We studied the coexistence of reported allergies in children who use asthma medication. Additionally, we tested the hypothesis that asthma severity is greater among children with certain combinations of co-morbid allergic conditions.

**Methods:**

For this cross-sectional study, 703 children (ages 4 to 12 years) from the PACMAN cohort study were selected. All of the children were regular users of asthma medication. The study population was divided into nine subgroups according to parental-reported allergies of the child (hay fever, eczema, food allergy or combinations of these). In order to assess whether these subgroups differed clinically, the groups were compared for child characteristics (age, gender, family history of asthma), asthma exacerbations in the past year (oral corticosteroids (OCS) use; asthma-related emergency department (ED) visits), asthma control, fractional exhaled nitric oxide level (FeNO), and antihistaminic usage.

**Results:**

In our study, 79.0 % of the parents reported that their child suffered from at least one atopic condition (hay fever, food allergy and eczema), and one quarter of the parents (25.6 %) reported that their child suffered from all three atopic conditions. Having more than one atopic condition was associated with an increased risk of OCS use (OR = 3.3, 95 % CI = 1.6 – 6.6), ED visits (OR = 2.3, 95 % CI = 1.2 – 4.6) in the past year and inadequate short term asthma control (OR = 1.9, 95 % CI = 1.3 – 2.8).

**Conclusions:**

Children who use asthma medication often also have other allergic conditions. Parental reported allergies were associated with a higher risk of more severe asthma (more asthma complaints and more asthma exacerbations).

## Background

Childhood allergic diseases have a major impact on a child’s quality of life, as well as that of their parents [1]. Therefore, it is important to have a better understanding of the risk factors associated with the development of asthma in children, as well as the factors associated with more severe asthma. The term “allergy” refers to a hypersensitivity reaction initiated by immunologic mechanisms, and although all people are continuously exposed to different allergens, only a limited group of individuals experience adverse immunologic mechanisms [2]. Persistent asthma is often treated with inhaled corticosteroids (ICS) in combination with short acting beta agonists (SABA) as needed, or sometimes in more severe cases, long acting beta agonists and/or leukotriene antagonists [3]. When asthma is controlled, there should only be occasional recurrence of symptoms, and severe asthma exacerbations should be rare [4]. One of the risk factors for asthma severity that has been identified is atopy [5, 6]. Atopic individuals are prone to developing allergic symptoms. Asthma, food allergies, eczema, and hay fever are common childhood atopic conditions with an increasing prevalence in the western world [7].

In general, eczema peaks in the child’s first years of life as an “entry point” for subsequent allergic disease, and consequently the prevalence of asthma and allergic rhinitis increases over time as sensitization develops [8].

Several studies have investigated the coexistence of food allergies and asthma, hay fever and asthma, or eczema and asthma [8–11]. However, most of these studies have only assessed the relationship between two conditions. They did not assess the effect of a combination of allergies, and they only focused on atopic patients. In this study, we examined the coexistence of allergies and the use of allergy related medication in a large cohort of children who use asthma medication and were recruited through community pharmacies. As a result of the inclusion of the participants from the community pharmacies, this cohort covered the whole spectrum of children with mild to severe asthma. Furthermore, we assessed the differences in the measurement of asthma severity among children with and without different allergies and combinations thereof.

## Methods

### Study population

At the time of this analysis, 744 children (ages 4 to 12 years) were included in the ongoing PACMAN (Pharmacogenetics of Asthma medication in Children: Medication with Anti-inflammatory effects) cohort study. Complete data on allergies was available for 703 children. The children were regular users (≥3 prescriptions in the last two years and ≥ 1 prescription in the last 6 months) of asthma medications (R03 on the ATC (Anatomical Therapeutic Chemical) coding system) and were recruited through community pharmacies in the Netherlands. The children and their parents were invited to their regular pharmacy for a study visit [12]. The design and rationale of the PACMAN study has been described elsewhere [12]. Data were collected with the help of pharmacists belonging to the Utrecht Pharmacy Practice Network for Education and Research (UPPER), and the work was conducted in compliance with the requirements of the UPPER institutional review board of the Department of Pharmacoepidemiology and Clinical Pharmacology, Utrecht University. The PACMAN study has been approved by the Medical Ethics Committee of the University Medical Centre Utrecht. Written, informed consent for all participants in the study was obtained from either the participants themselves, or, where participants were minors, a parent or guardian [12].

### Data collection

The parents completed a questionnaire during the pharmacy visit. The questionnaire contained questions regarding general health, asthma and respiratory symptoms, allergy symptoms, medication use, adherence to medication (Medication Adherence Rating Scale (MARS) questionnaire [13]), socio-demographic factors, and asthma symptoms. In addition, the child’s fractional exhaled nitric oxide level (FeNO) was measured with a handheld analyzer (Niox Mino, Aerocrine, Solna, Sweden).

To measure co-morbid atopic conditions, parents were asked: Has your child ever had a food allergy (FA) (itching, rash/hives, vomiting, diarrhea, runny nose, sneezing, stuffiness and cough)? Has your child ever had eczema? Has your child ever had hay fever (HF)?

The use of oral corticosteroids (OCS) and the amount of emergency department (ED) visits were used to measure asthma severity. Furthermore, the Dutch version of the 6-item Asthma Control Questionnaire (ACQ) was applied to assess current asthma control. ACQ ≥ 1.5 was used as a cut-off value indicating poorly controlled asthma [14].

### Statistical analyses

**Fig. 1:**
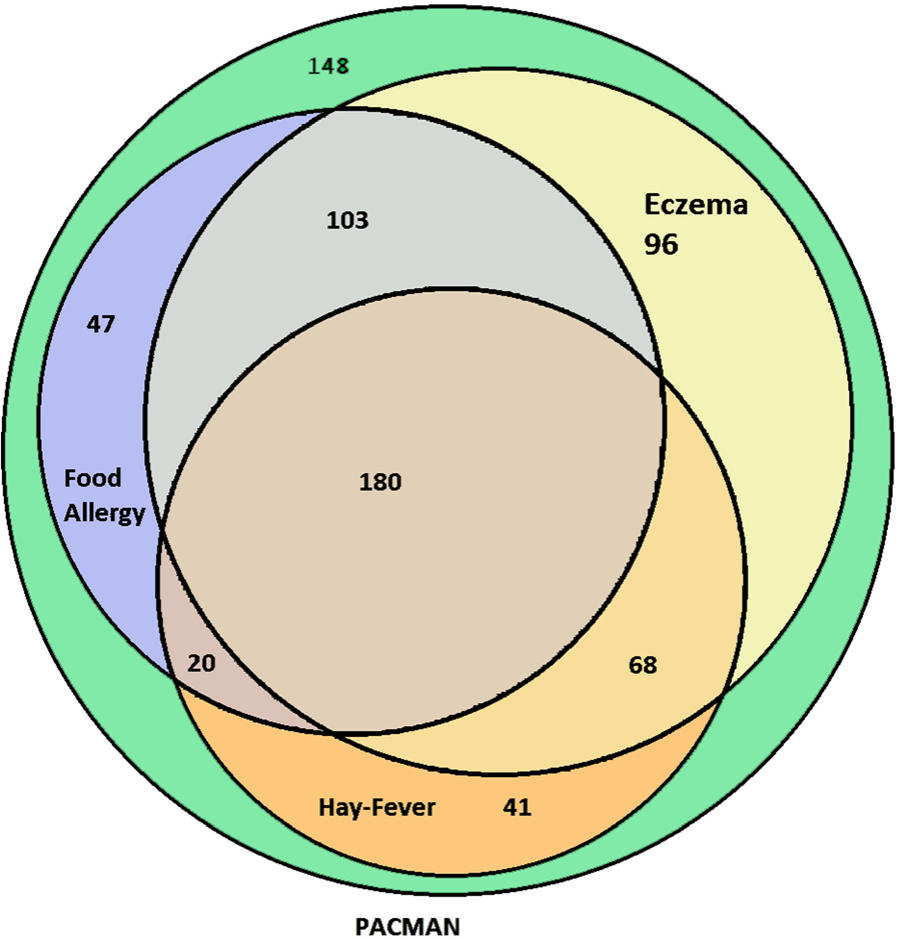
The co-existence of allergies in the study population

The study was a cross-sectional analysis in the baseline measurements of the PACMAN cohort study. The study population was stratified into nine subgroups according to the allergies that the parents had reported. The first three groups reported HF, FA, or eczema irrespective of whether or not they had also reported one or more of the other studied allergies. Then all possible combinations of allergies were defined (FA + eczema, eczema + HF, FA + HF, FA + eczema + HF) (see Fig. 1 and Table 1).

**Table 1:** Characteristics and antihistamines usage

The population in the Venn diagram ^a^		Number (Percentage)	Mean Age ± SD	Median FeNO
			(P Value)^b^	(P Value)^c^
				[IQR]
	Study Population	703	8.4 ± 2.4	13.0 [7.0 – 27.0]
	Without history of allergies	148 (21.1 %)	8.1 ± 2.4 (.104)	11.0(.084) [6.0 – 27.0]
	Eczema	447 (63.6 %)	8.5 ± 2.5 (.485)	13.0 (.222) [8.0 – 26.0]
	Food allergy	350 (49.8 %)	8.4 ± 2.5 (.695)	13.0 (.294) [8.0 – 27.0]
	Hay fever	309 (44.0 %)	8.9^d^ ± 2.3 (.000)	15.0^d^ (.005) [8.0 – 29.8]
	Food allergy + Eczema	283 (40.3 %)	8.5 ± 2.5 (.646)	14.0(.072) [8.3 – 27.8]
	Eczema + Hay fever	248 (35.3 %)	8.8^d^ ± 2.4 (.002)	15.0^d^ (.036) [8.5 – 27.5]
	Food allergy + Hay fever	200 (28.5 %)	8.7^d^ ± 2.4 (.035)	15.0 (.153) [8.0 – 28.0]
	Food allergy + Eczema + Hay fever	180 (25.6 %)	8.8^d^ ± 2.4 (.035)	15.0 (.099) [9.0 – 27.0]
	At least two allergies	371 (52.8 %)	8.6 ± 2.4 (.109)	14.0^d^ (.029) [8.0 – 28.0]

^a^For a larger diagram see Fig. 1

^b^With independent samples T-test

^c^With Mann–Whitney test

^d^P Value < 0.05

The characteristics and asthma severity measures of these groups (age, gender, family history of asthma, breast feeding, FeNO, use of allergy medications, OSC usage, ED visits and ACQ) were compared between the groups of children with and without specific combination of atopic conditions (colored area in the first column of Table 1 and the rest of PACMAN population).

We used the independent samples T-test and the Chi-Square test where appropriate. As the distribution of FeNO was not normal, according to the Kolomogorov-Smirnov and the Shapiro-Wilk test, the Mann–Whitney test was used to compare median FeNO between different groups. Logistic regression was applied for multivariate analyses. Age, gender and use of antihistamines were considered potential confounding factors. The potential confounding factors were included in the multivariate model. The Odds Ratios (OR) for OCS use, ED visits and ACQ were adjusted for age and gender and reported with 95% confidence intervals CI). Adjusting the OR for the use of antihistamines and adherence to therapy did not change the results (Table 4).

## Results

### Co-existence of allergies

In the study population, 79.0% (555/703) of the parents reported that their children had suffered from at least one of the assessed allergies. Eczema was the most common condition (63.6 %). Almost half of the study population reported a history of food allergy (49.8 %), and hay fever was reported by 44.0 %. 25.6 % (180/703) of the participants reported symptoms of all three allergies (food allergy, eczema and hay fever), while 21.1 % did not report any of these symptoms. (See Fig. 1 and Table 1).

### Baseline characteristics

Characteristics of the study population are shown in Table 2.

**Table 2:** Characteristics of study population

Study population (*n* = 703)	
General characteristics	
Male gender, %	62.0
Age, mean ± SD	8.4 ± 2.5
Clinical characteristics	
Parental-reported Eczema, %	63.6
Parental-reported Food Allergy, %	49.8
Parental-reported Hay fever, %	44.0
Asthma family history ( One or more parents with history of asthma), %	48.0
Antihistamine usage, %	30.6
ICS usage, %	87.8
SABA usage, %	84.8
LABA usage, %	23.5
LTRA usage, %	8.8
Breast fed, %	74.9
Median FeNO (IQR)	13.0 (7.0 – 27.0)
OCS usage in the past year, %	7.0
Asthma-related ED visit in the past year, %	6.3

**Fig. 2:**
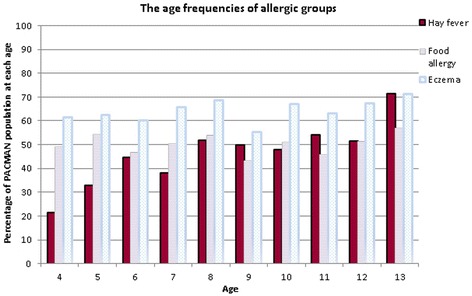
The age frequencies of allergic groups in the study population

The trends of the main allergic groups’ age distributions are shown in Fig. 2. For hay fever an ascending trend is visible (Fig. 2). The mean age of the study population was 8.4 years. However, the mean age of the subgroup of children that reported having hay fever (irrespective of whether they had other allergies) was significantly higher (8.9 years, *p* < 0.001) (Table 1). Also, the occurrence of hay fever increased from almost 20 % in the 4-year-olds to more than 50 % in the 12-year-olds (Fig. 2). The frequency of children with a positive asthma family history (father or mother) in the total studied PACMAN population was 48.0 %. In the subgroup of children who reported having had hay fever, there was an even higher risk of a family history of asthma (55 %) compared to the children who did not report having had hay fever (45.0 %) (OR = 1.7 95 % CI = 1.2 – 2.3). Furthermore, in the subgroup of children with a reported food allergy, there was a trend towards a higher risk of a family history of asthma (51.2 % to 48.8 %, OR = 1.3 95 % CI = 1.0 - 1.8) (Table 3). The median of FeNO in the study population was 13.0 (Interquartile Range (IQR) = 7.0 – 27.0). The children who reported having had hay fever had a significantly higher FeNO (median = 15.0, IQR = 8.0 – 29.8, *p* < 0.01) (Table 1). Gender or having been breastfed did not significantly differ between allergic subgroups.

**Table 3:** Differences in asthma family history in the allergic subgroups

	Asthma family history % (P Value)		Odds Ratio (95 % CI)
Group	Present	Not present	
Study population	48.0		
Without history of allergies	44.0 (.230)	56.0	0.8 (0.5 – 1.2)
Eczema	48.5 (.741)	51.5	1.1 (0.8 – 1.4)
Food allergy	51.2 (.096)	48.8	1.3 (1.0 - 1.8)
Hay fever	55.0^a^ (.001)	45.0	1.7^a^ (1.2 – 2.3)
Food allergy + Eczema	51.5 (.137)	48.5	1.3 (0.9 – 1.7)
Eczema + Hay fever	54.8^a^ (.009)	45.2	1.5^a^ (1.1 – 2.1)
Food allergy + Hay fever	57.7^a^ (.001)	42.3	1.7^a^ (1.2 – 2.4)
Food allergy + Eczema + Hay fever	57.5^a^ (.004)	42.5	1.7^a^ (1.2 – 2.4)
At least two allergies	51.3 (.070)	48.7	1.3 (1.0 – 1.8)

^a^P Value < 0.05 with chi-square test

### Oral antihistaminic drug usage

Oral antihistaminic drugs were used by almost 30 % of the study population. The top three oral antihistaminic drugs (Loratadine, Cetirizine and Fenistil) were equally distributed among all the allergy subgroups.

### Asthma outcomes

Severity of asthma was assessed by OCS usage, ED visits and ACQ using both univariate and multivariate analyses. 9.1 % of the children who reported eczema symptoms used OCS (Table 4). This was significantly higher when compared to the use of OCS in the non-eczema population (3.2 %) (OR = 3.0, 95 % CI = 1.4 – 6.6). The use of OCS for the subgroup that had symptoms of food allergy was 9.6 %; this was also statistically significantly different compared to 4.3 % of the non-food allergy population (OR = 2.3, 95 % CI = 1.2 – 4.4). There was a trend towards a higher risk for the use of OCS in all allergy subgroups. However, the group of children who did not report a history of allergic conditions did not have an increased risk for the use of OCS (Table 4).

**Table 4:** Differences in outcomes of each subgroups in whole study population

	OCS usage % (P Value)	Univariate analysis	Multivariate analysis ^b^	E.D visit in past year % (P Value)	Univariate analysis	Multivariate analysis ^b^	Poorly controlled refer to ACQ-6 % (P Value)	Univariate analysis	Multivariate analysis ^b^
		OR	OR		OR	OR		OR	OR
		(95 % CI)	(95 % CI)		(95 % CI)	(95 % CI)		(95 % CI)	(95 % CI)
Study population	7.0			6.3			18.2		
Without history of allergies	4.1 (0.12)	0.5 (0.2–1.2)	0.5 (0.2–1.2)	4.1 (.215)	0.6 (0.2–1.4)	0.5 (0.2–1.3)	14.3 (.118)	0.7 (0.4–1.1)	0.7 (0.4–1.1)
Eczema	9.1^a^ (.003)	3.0^a^ (1.4–6.6)	3.0^a^ (1.4–6.6)	8.1^a^ (.010)	2.7^a^ (1.2–5.9)	2.7^a^ (1.2–6.0)	20.4 (.053)	1.5 (1.0–2.3)	1.5^a^ (1.0–2.4)
Food allergy	9.6^a^ (.007)	2.3^a^ (1.2–4.4)	2.3^a^ (1.2–4.4)	8.0 (.068)	1.8 (1.0–3.4)	1.8 (0.9–3.4)	21.3^a^ (.039)	1.5^a^ (1.0–2.2)	1.5^a^ (1.0–2.2)
Hay fever	8.0 (0.36)	1.3 (0.7–2.4)	1.4 (0.8–2.5)	6.0 (.765)	0.9 (0.5–1.7)	1.1 (0.6–2.1)	22.7^a^ (.007)	1.7^a^ (1.2–2.5)	1.8^a^ (1.2–2.7)
Food allergy + Eczema	11.6^a^ (.000)	3.2^a^ (1.7–6.0)	3.3^a^ (1.8–6.1)	9.6^a^ (.005)	2.4^a^ (1.3–4.6)	2.5^a^ (1.3–4.7)	22.1^a^ (.028)	1.5^a^ (1.0–2.3)	1.6^a^ (1.1–2.3)
Eczema + Hay fever	9.5 (.056)	1.8 (1.0–3.2)	1.8^a^ (1.0–3.3)	7.1 (.557)	1.2 (0.6–2.3)	1.4 (0.7–2.7)	24.5^a^ (.002)	1.9^a^ (1.3–2.8)	1.9^a^ (1.3–2.9)
Food allergy + Hay fever	9.2 (0.14)	1.6 (0.9–2.9)	1.6 (0.9–3.0)	6.7 (.776)	1.1 (0.6–2.2)	1.2 (0.6–2.5)	25.4^a^ (.002)	1.9^a^ (1.3–2.8)	1.9^a^ (1.3–2.9)
Food allergy + Eczema + Hay fever	10.3^a^ (.045)	1.9^a^ (1.0–3.4)	1.9 (1.0–3.6)	7.5 (.467)	1.3 (0.7–2.5)	1.5 (0.7–2.9)	25.3^a^ (.005)	1.8^a^ (1.2–2.7)	1.9^a^ (1.2–2.8)
At least two allergies	10.1^a^ (.001)	3.2^a^ (1.6–6.4)	3.3^a^ (1.6–6.6)	8.4^a^ (.020)	2.2^a^ (1.1–4.3)	2.3^a^ (1.2–4.6)	22.4^a^ (.003)	1.9^a^ (1.2-2.8)	1.9^a^ (1.3–2.8)

The referent group for all these odds ratios is the entire study population

^a^ *P* Value < 0.05 with logistic regression test

^b^Adjusted for age and gender

Emergency department visits during the past year were significantly higher (8.1 %, OR = 2.7, 95 % CI = 1.2 – 6.0) in the population who had a history of eczema as compared to the rest of the population (3.2 %) (Table 4).

The Asthma Control Questionnaire (ACQ) was assessed in all the defined groups, and 18.2 % of the total study population suffered from poorly controlled asthma. The frequencies of poorly controlled asthmatics in all allergic subgroups were significantly higher (*p* < 0.05) as compared to the non-allergic population. They were 21.3 %, 20.4 % and 22.1 % in the populations with a history of eczema, food allergy or both, respectively. The frequencies of poorly controlled patients were even higher in all the subgroups that reported hay fever (22.7 % - 25.4 %) or more than one allergy (22.4 %) compared to the rest of study population (Table 4).

## Discussion

In this large pharmacy-based study of children with a reported use of asthma medication, we found that the prevalence of children that reported symptoms of one or more allergy syndromes was high, and patients that reported more atopic conditions had a greater odds of more severe asthma.

In general, children with asthma and co-morbid allergic conditions were more often poorly controlled compared to their non-allergic peers. Furthermore, usage of OCS and asthma-related ED visits were more common in children who reported more than one atopic condition, which was approximately half of the study population. This indicates that the presence of a more complicated allergic phenotype significantly influences the severity of asthma [15].

To our knowledge, there is limited research that has studied the association of allergic comorbidities and asthma severity [16]. However several longitudinal studies have shown that approximately half of eczema patients will develop asthma, particularly patients with severe eczema [8]. A study by Roberts et al. showed that children with food allergies are around 6 times more likely to suffer from severe asthma later in life than children who did not have food allergies. Similarly, Priftis et al. showed that approximately 40 % of children who were diagnosed with an egg and/or fish allergy in the first three years of their life reported current asthma symptoms at school age [17, 18]. Moreover, hay fever has been described as a major risk factor for asthma [19, 20]. In the current study, eczema was the most frequently reported allergy among the three allergies (food allergy, eczema and hay fever), reported by 63 % of the population (Table 2). A remarkably high percentage of the parents (25.6 %) reported that their children had experienced all three allergies (Fig. 1). The prevalence of food allergy in the current study was also very high (49.8 %). Earlier studies showed that the prevalence of food allergy varied between 3 % and 35 % [7]. Likewise a Dutch study reported a prevalence of (current) self-reported food allergy around 7.2 % among school children in the Netherlands [21]. The high prevalence in our study may have been influenced by the fact that we asked whether the child had ever experienced symptoms. Some children might have only experienced symptoms in early childhood, and this may have caused a larger prevalence than the prevalence of current food allergy symptoms. Nevertheless, we do realize that self-reporting might lead to an overestimation. Unfortunately, data regarding provocation testing to confirm an actual diagnosis of food allergy were not available. However, it has been shown that results from screening questionnaires, comparable to the one we used in this study, were in concordance with results from specific IgE measurements and information obtained from patient records [22, 23].

When we assessed the effect of age on the development of allergic disease, we noticed that the occurrence of hay fever increased with age in our study population (Fig. 2). Moreover, the mean age of the hay fever group (8.9 ± 2.5) was significantly higher than the mean age in the overall study population (Table 2). The same trend was reported by Spergel et al. where the incidence of hay fever increased over time during childhood. This might be caused by sensitization developed through other allergic conditions [8]. Ghouri et al. showed an increase in the prevalence of hay fever during childhood in England as well [24]. On the other hand, age trends in the occurrence of the eczema were not observed. Spergel et al. reported age incidence of eczema peaks in the first years of life [8]. It might, therefore, be that our population was too old to observe this trend. The median FeNO level was significantly higher in the hay fever group. This is in alignment with other studies that confirm high FeNO levels in hay fever sufferers [25, 26].

Our study was limited by the lack of physicians’ diagnoses on allergic diseases or objective immunological test results. We used a questionnaire to obtain information about the history of allergic conditions. Other studies (such as ISAAC [27]) have also used questionnaire data. We realize, however, that this questionnaire data might differ from objective tests, and the occurrence of allergic diseases might therefore have been overestimated due to the use of parental-reported data. However, the strength of our study is in the selection of a large set of asthmatic children through community pharmacies. Our population represents a cross-section of the everyday pediatric asthma population that varies in the severity of the disease, health care utilization and asthma control.

## Conclusions

In conclusion, our study suggests that children with asthma and co-morbid atopic conditions are at risk for more exacerbations and less well-controlled asthma in comparison to children who did not report allergies. The children who were reported to have had more than one allergic co-morbidity were especially at risk of having less well controlled asthma and more severe exacerbations. This may have clinical implications, such as more unscheduled health care visits and hospitalizations, as these patients may experience more severe asthma. These children should be carefully monitored and might benefit from asthma/allergy specialist care at an earlier stage.

## Abbreviations

ACQ: Asthma Control Questionnaire
ATC: Anatomical Therapeutic Chemical
CI: confidence interval
ED: emergency department
FA: food allergy
FeNO: fractional exhaled nitric oxide level
HF: hay fever
ICS: Inhaled Corticosteroids
IQR: Interquartile Range
MARS: Medication Adherence Rating Scale
OCS: oral corticosteroids
OR: Odds Ratios
PACMAN: Pharmacogenetics of Asthma medication in Children: Medication with Anti-inflammatory effects
SABA: short acting beta agonists
UPPER: Utrecht Pharmacy Practice Network for Education and Research
